# Emergency Providers’ Pain Management in Patients Transferred to Intensive Care Unit for Urgent Surgical Interventions

**DOI:** 10.5811/westjem.2018.7.37989

**Published:** 2018-08-08

**Authors:** Quincy K. Tran, Tina Nguyen, Gurshawn Tuteja, Laura Tiffany, Ashley Aitken, Kevin Jones, Rebecca Duncan, Jeffrey Rea, Lewis Rubinson, Daniel Haase

**Affiliations:** * University of Maryland School of Medicine, Department of Emergency Medicine, Baltimore, Maryland; † Johns Hopkins University, Baltimore, Maryland; ‡ University of Maryland at College Park, College Park, Maryland; § The R. Adam Cowley Shock Trauma Center, Critical Care Resuscitation Unit, Baltimore, Maryland; ¶ The R. Adam Cowley Shock Trauma Center, University of Maryland School of Medicine, Program of Trauma, Baltimore, Maryland

## Abstract

**Introduction:**

Pain is the most common complaint for an emergency department (ED) visit, but ED pain management is poor. Reasons for poor pain management include providers’ concerns for drug-seeking behaviors and perceptions of patients’ complaints. Patients who had objective findings of long bone fractures were more likely to receive pain medication than those who did not, despite pain complaints. We hypothesized that patients who were interhospital-transferred from an ED to an intensive care unit (ICU) for urgent surgical interventions would display objective pathology for pain and thus receive adequate pain management at ED departure.

**Methods:**

This was a retrospective study at a single, quaternary referral, academic medical center. We included non-trauma adult ED patients who were interhospital-transferred and underwent operative interventions within 12 hours of ICU arrival between July 2013 and June 2014. Patients who had incomplete ED records, required invasive mechanical ventilation, or had no pain throughout their ED stay were excluded. Primary outcome was the percentage of patients at ED departure achieving adequate pain control of ≤ 50% of triage level. We performed multivariable logistic regression to assess association between demographic and clinical variables with inadequate pain control.

**Results:**

We included 112 patients from 39 different EDs who met inclusion criteria. Mean pain score at triage and ED departure was 8 (standard deviation 8 and 5 [3]), respectively. Median of total morphine equivalent unit (MEU) was 7.5 [5–13] and MEU/kg total body weight (TBW) was 0.09 [0.05–0.16] MEU/kg, with median number of pain medication administration of 2 [1–3] doses. Time interval from triage to first narcotic dose was 61 (35–177) minutes. Overall, only 38% of patients achieved adequate pain control. Among different variables, only total MEU/kg was associated with significant lower risk of inadequate pain control at ED departure (adjusted odds ratio = 0.22; 95% confidence interval = 0.05–0.92, p = 0.037).

**Conclusion:**

Pain control among a group of interhospital-transferred patients requiring urgent operative interventions, was inadequate. Neither demographic nor clinical factors, except MEU/kg TBW, were shown to associate with poor pain management at ED departure. Emergency providers should consider more effective strategies, such as multimodal analgesia, to improve pain management in this group of patients.

## INTRODUCTION

Although pain is the most common complaint for emergency department (ED) visits,^1^,^2^ and the fifth vital sign,^3^ inadequate treatment of pain occurs frequently in the ED: up to 74% of patients are discharged with moderate to severe pain^4^ and 57% of patients fail to achieve a 50% pain score reduction.^5^ Pain is a major cause of patient dissatisfaction in United States healthcare.^6^ In 2012, the Centers for Medicare and Medicaid instituted the new value-based purchasing program, which tied financial incentives with higher patient satisfaction scores^7^ and measures to improve patient care, including pain management.^7^ Therefore, effective pain management has become an important aspect of patient care.

Pain undertreatment is multifactorial; it has been linked to providers’ concern for drug-seeking behaviors^8^ and physicians’ perception that pain was exaggerated.^9^ However, patients with objective findings of pain, such as long bone fractures, were twice as likely to receive opioid pain medication^10^ than those who did not have fractures. Therefore, our goal was to study pain management among a group of patients who were inter-hospital transferred to intensive care units (ICU) for urgent surgical interventions and to identify any demographic or clinical factors associated with pain undertreatment. We hypothesized that these patients who showed objective findings for their pain complaints would receive adequate pain control at ED departure.

## METHODS

### Patient Selection

We performed a retrospective study using a convenience sample of non-traumatic, interhospital adult patients who were transferred from a referring ED to any ICU at a quaternary, academic center for urgent surgical interventions, defined as having surgery within 12 hours of arrival. We included interhospital-transferred patients from referring EDs between July 2013 to June 2014 who were taken to the operating room urgently within 12 hours of arrival. We excluded patients who required mechanical ventilation in the ED and ED records missing pain assessment at ED triage or departure. We also excluded patients who reported no pain throughout their ED stay. The study was approved by our institutional review board.

### Outcome

Primary outcome was percentage of patients receiving adequate pain control at ED departure. Adequate pain control was defined *a priori* as pain level, reported on 11-point verbal scale (0–11), at ED departure that was equal or less than 50% from triage levels. Pain reduction at equal or less than 50% was considered clinically meaningful to ED patients in previous studies.^5^,^11^ We calculated percentage of pain reduction at ED departure as (pain level of departure / pain level at triage) x 100. If pain score at ED departure was equal or greater than triage pain score, percentage of pain reduction would be assigned 100%. Secondary outcomes included percentage of adequate pain control by admitting medical services, and predictors that may have been associated with inadequate pain control at ED departure.

**Population Health Research Capsule**

What do we already know about this issue?

Pain management in the emergency department has been inadequate, but patients with objective findings for pain were more likely to receive pain medicine.

What was the research question?

Whether patients, who were transferred to a quaternary academic center for urgent surgical intervention, would receive adequate pain management.

What was the major finding of the study?

Pain management among patients with surgical pathology was inadequate. Emergency providers (EPs) did not employ effective strategies for pain management in these patients.

How does this improve population health?

EPs should employ effective strategies, including multimodal analgesia, to improve pain management among patients who needed surgical interventions.

### Data Collection and Analysis

Investigators (TN, GT, LT, AA, RD, KJ, JR, DH), who were not blinded to the study hypothesis, were first trained by the principal investigator (PI) (QKT) for data extractions. Data were extracted to a standardized Microsoft Access form (Microsoft Corp, Redmond, Washington) and 40% was randomly reviewed by the PI to maintain interrater agreement of at least 90% for systolic blood pressure, heart rate, and pain at ED triage, ED departure and medication administration. The team met every month to discuss data extraction issues and to adjudicate disagreements between junior investigators and the PI until data collection was completed.

ED records from patients who were taken to the operating room within 12 hours of ICU arrival were identified and examined for data extraction. Independent variables extracted from patients’ ED records included demographic factors (age, gender, triage day of week, triage time of day, admitting services); clinical data (Emergency Severity Index [ESI]; vital signs/pain score at ED triage and departure; ED length of stay [LOS]; components for the sequential organ failure assessment score [SOFA]; presence of continuous infusion; and dosage of pain medication). The continuous infusion did not include vasopressors, which were part of the SOFA score. To evaluate the opioid dose that patients received, we converted the doses of the different parenteral or intravenous (IV) opioids to morphine equivalent unit (MEU), as previously described.^12^,^13^ We considered 0.15 milligrams (mg) of IV hydromorphone and 0.01 mg of IV fentanyl as 1 MEU. Similarly, 5mg of oral oxycodone, hydrocodone were equivalent to 2 MEU.^12^,^13^

Data were expressed as mean and standard deviation (SD) or median with interquartile range (IQR), where appropriate. The effect of variables on inadequate pain control was assessed using multivariable logistic regression. We *a priori* selected therapeutic interventions, such as total MEU, MEU per kilogram (kg), and time interval to first narcotics, that may have clinically affected outcomes, to be included in the multivariable logistic regression. Independent variables were first evaluated in univariable analyses; we excluded those with weak association with outcome variable (p-value≥0.101) from the multivariable logistic regressions. All p-value ≤0.05 were considered statistically significant. We performed all statistical analyses using Sigma Plot version 14 (Systat Software, San Jose, California).

## RESULTS

### Patient Characteristics

**Figure 1:**
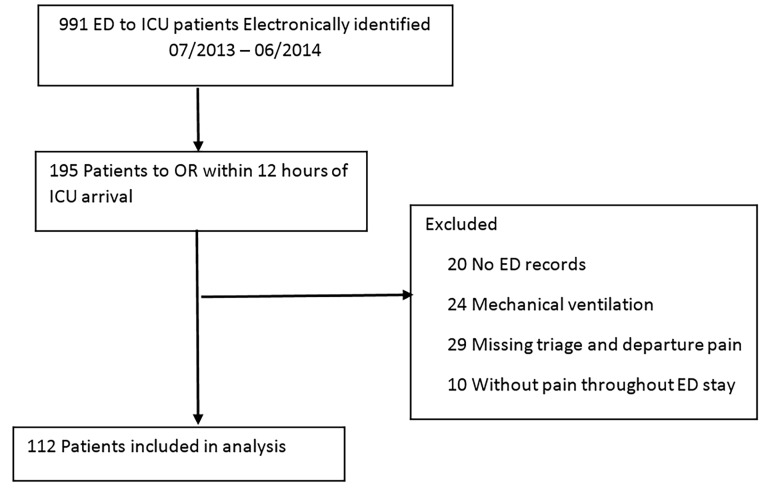
Patient selection diagram. *ED*, emergency department; *ICU*, intensive care unit; *OR*, operating room.

We electronically identified 195 patients who were transferred to any adult ICU at our institution between July 2013 and June 2014; 112 patients who were transferred from 39 unique EDs met inclusion criteria and were included in the final analysis (Figure 1). The majority of patients were male (63%), and mean age was 57 (SD = 18) years. Median ED LOS was 3.9 [2.5–6.4] hours. Median [IQR] of ESI and SOFA scores for the patients were 3 [2–3] and 1 [0–3], respectively. The mean pain score at triage was 8 (3) with a majority of patients (66%) reporting a severe pain score from 8–10. The admitting service with the most patients in our study was vascular surgery (24%), while acute care emergency surgery (ACES) and cardiac surgery were second (18%) and third (17%), respectively.

### Interventions by Emergency Providers (EP)

Most patients (66%) received only narcotics, and the median number of administered doses was 2 [1–2]. The median number of pain assessment was 3 [2–5]. The majority of administered pain medications were narcotics (66%), while the time interval from triage to first dose of narcotics was 61 (35–177) minutes. Median of total MEU administered was 7.5 [5–13] and median of total MEU per body weight was 0.09 [0.05–0.16] MEU/kg body weight (Table 1).

### Outcomes

Overall, emergency providers (EP) poorly managed pain in this high-risk group of patients. Pain control was adequate in only 38% of patients and inadequate in 62% (Table 2A), with the mean pain level at ED departure 5 (3). Vascular surgery had the highest percentage (59%) of patients receiving adequate pain control, among the five major admitting services. Among the independent variables, only ESI was significantly associated with inadequate pain control (OR = 8.29, 95% confidence interval [CI] = 1.01–68.1, p=0.049) (Table 2B). However, in the multivariable logistic model (Table 2B), only total MEU/kg body weight was associated with adequate pain control (OR = 0.22, 95% CI = 0.05–0.92, p = 0.037).

We also performed a subgroup analysis of patients presenting with severe pain (triage pain 8–10) only, to avoid the confounding factor of whether the patient refused pain treatment. There were 74 (66%) patients presenting with severe pain. Thirty patients (41%) had adequate pain control at ED departure. Comparing to all patients, median of total MEU (8 [5–14], p=0.3) or MEU/kg of body weight (0.11 [0.06–0.17], p = 0.14) was not significantly different. Multiple logistic regression adjusting for the same clinically-significant factors showed that no interventions by EPs were associated with adequate pain control (Table 2B).

## DISCUSSION

Our study showed that among patients who were transferred from EDs to ICUs for urgent surgical interventions, only 38% achieved meaningful reduction of pain level at ED departure, defined as 50% or less of triage levels. We identified one factor, total MEU/kg total body weight, associated with adequate pain control. However, we identified three potential barriers for adequate pain management in the EDs. Our findings suggested that pain control for this group of ED patients with time-sensitive diseases was inadequate.

In our study, the median time interval from triage to IV morphine was 61 minutes (range 35–177). A previous prospective, multicenter study showed that the median time from ED triage to any analgesic administration for ED patients presenting with moderate to severe pain^4^ was 90 minutes (range 0–962 minutes). Only 29% of patients who were given analgesics received them within one hour of arrival. Although the results from these two studies may not be comparable, our study suggested a shorter time from triage to administration of pain medication and that 50% of the patient population received pain medication within one hour of arrival (data not shown). These results suggested that EPs in our study did recognize the distress of their patients and tried to relieve their discomfort, but their efforts appeared inadequate.

Pain management has been shown to be poor in the ED.^5^,^11^ Reasons for inadequate pain control in EDs may result from EPs’ misconception of compromising a patient’s mental status^8^ or clouding physical examination in surgical patients^14^ such as patients in our population. In addition to these possible barriers, our study identified three additional potential barriers for adequate pain control. First, the amount of total MEU that our patients received was less than the recommended 0.1mg/kg body weight,^15^ although even this dosage of 0.1mg/kg morphine was inadequate in relieving severe pain.^15^ A second potential barrier was from the EPs’ practice of giving patients in our study multiple smaller doses of pain medication. Administering multiple doses of pain medication was not associated with adequate pain control in a randomized study by Chang et al.,^16^ comparing to a single dose of 2mg hydromorphone, which is equivalent to 14 MEU. According to this study, one single dose of 2mg of hydromorphone was significantly associated with higher percentage of adequate pain control, comparing to multiple smaller dosage of pain medication, while achieving similar safety profiles. Therefore, EPs should consider giving patients with clear pathology for pain higher initial doses of pain medication to achieve levels higher than 0.1 mg/kg body weight.

Our study also identified the lack of multimodal analgesia among our patient population, who only received either narcotic or nonsteroidal anti-inflammatory drug (NSAID) pain medication. A recent study showed that a combination of ibuprofen and acetaminophen was equally effective as a single dose of opioids among patients with severe extremity pain.^17^ Furthermore, other analgesic modalities such as regional analgesia, gabapentinoids, and/or the N-methyl-D-aspartate class of glutamate receptor antagonists (tramadol, nitrous oxide) have been shown to be effective adjuncts to narcotic analgesia.^18^ Therefore, more education to increase awareness and comfort about using other analgesic modalities for pain management will improve patients’ pain relief.^19^

## LIMITATIONS

Our study had several limitations. First, we did not have a control group to compare the efficacy of pain management in non life-threatening situations to these patients who would need transfer for urgent surgical intervention. Secondly, we did not assess the effect of ethnicity on inadequate pain control among our group of critically ill patients. A previous study suggested that African-American patients who were not taking opioids at home were less likely to achieve a 50% pain score reduction than other patients, even with similar analgesic dosage.^5^

Our study consisted of a heterogenous group of patients with a limited sample size; therefore, it did not allow us to investigate which disease states would be at higher risk for inadequate pain control. Although patients included in our study required urgent surgical intervention, the sample size of mortality was low (9%), which did not allow us to examine the association of poor analgesia with outcome, as refractory pain was shown to be associated with poor outcomes in some disease states such as aortic dissection.^20^ Furthermore, we excluded a group of patients requiring invasive mechanical ventilation, who had been shown to be at higher risk of not receiving analgesia in the ED.^21^ Finally, we were not able to assess whether patients took medication prior to presenting to the ED or whether patients refused pain medication in the ED, which would affect the amount of pain medication administration and overall effectiveness of pain management.

## CONCLUSION

Pain control among a group of interhospital-transferred patients requiring urgent operative interventions, was inadequate. No demographic or clinical factors, except total morphine equivalent unit per kg body weight, was associated with adequate pain management at ED departure. Emergency providers should consider a more effective strategy, such as multimodal analgesias, to improve pain management in this group of patients.

## Supplementary Information



## Figures and Tables

**Figure 2ab:**
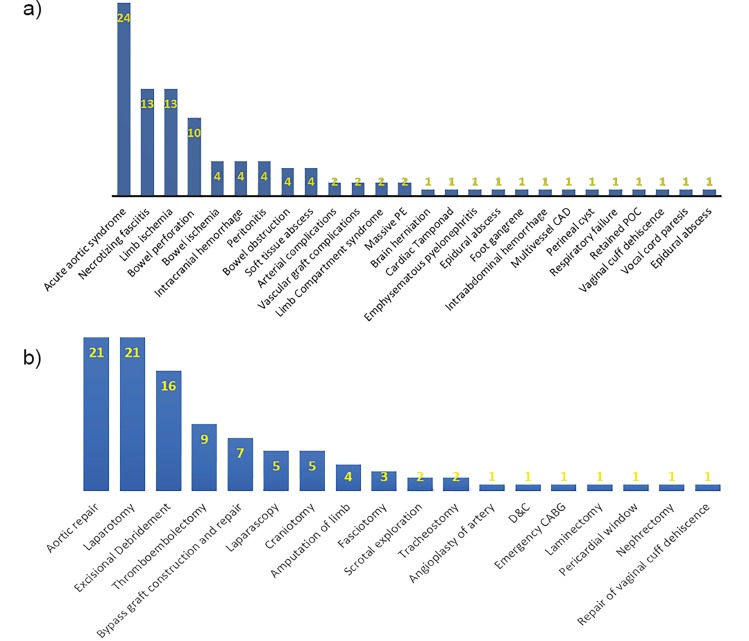
a) Categories of diagnoses among patients transferred for immediate surgical interventions; b) Categories of surgical procedures for transferred patients requiring urgent surgical interventions. Y-axis represented percentage of total population; X-axis represented names of categories. Acute aortic syndrome included type A and type B aortic dissection, aortic aneurysm, intramural hematoma, etc. PE, pulmonary embolism; CAD, coronary artery disease; POC, product of conception; Dand C, dilation and curettage; CABG, coronary artery bypass graft.

**Table 1:** Demographic information from 112 patients who were transferred to an Intensive care unit at a tertiary referral academic center and underwent surgical interventions within 12 hours of arrival.

Total patients, N (%)	112 (100)
Gender	
Male (N, %)	70 (63)
Female (N, %)	42 (37)
Age (years), mean (SD)	57 (18)
18–40 (N, %)	22 (20)
41–60 (N, %)	42 (38)
>61 (N,%)	48 (42)
Teaching hospital status,	
Non-teaching (N, %)	82 (73)
Teaching (N, %)	30 (27)
Ground travel distance (km)	51 [22–11]
≤20, (N, %)	24 (21)
20.1–50, (N, %)	25 (22)
50.1–100, (N, %)	30 (28)
≥100.1, (N, %)	33 (29)
ESI, median [IQR]	3 [2–3]
1–2 (N, %)	5 (4)
3 (N, %)	103 (92)
4–5 (N, %)	4 (4)
SOFA score, median [IQR]	1 [0–3]
0–1 (N, %)	63 (56)
2–5 (N, %)	43 (38)
>6 (N, %)	6 (6)
Triage systolic blood pressure, mean (SD)	134 (37)
≤89 mm Hg (N, %)	13 (12)
90–179 mm Hg (N, %)	84 (75)
≥180 mm Hg (N, %)	15 (13)
Triage heart rate, mean (SD)	92 (24)
≤59 bpm, N (%)	7 (6)
60–99 bpm, N (%)	64 (57)
≥100 bpm, (%)	41 (37)
Triage pain score, mean (SD)	8 (3)
0–3 (N, %)	8 (7)
4–7 (N, %)	30 (27)
8–10 (N, %)	74 (66)
Presence of continuous infusion, N (%)	
Yes	32 (29)
No	80 (71)
ED length of stay (hours), median [IQR]	3.9 [2.5–6.4]
Transport type, N (%)	
Air	38 (34)
Ground	74 (66)
Medical admitting services, N (%)	
ACES	20 (18)
Cardiac surgery	19 (17)
Neurosurgery	10 (9)
Soft tissue surgery	16 (14)
Vascular surgery	27 (24)
Other	20 (18)
Number of pain assessment, median [IQR]	3 [2–5]
≤3 (N, %)	64 (57)
≥4 (N, %)	48 (43)
Number of pain medication administration, median [IQR]	2 [1–3]
Pain medication type, N (%)	
No medication	27 (24)
NSAIDS only	2 (2)
Narcotics only	74 (66)
Narcotics and NSAIDs	9 (8)
Total MEU, median [IQR]	7.5 [5–13]
MEU per Kg median, [IQR]	0.09 [0.05–0.16]
Time to first NSAIDs (min), median [IQR]	55 [22–113]
Time to first narcotic (min), median [IQR]	61 [35–177]
ICU arrival – operations (min), median [IQR]	195 [121–422]
Hospital LOS (day), median [IQR]	9 [6–16]
Mortality, N (%)	10 (9)

*N*, number of patients; *SD*, standard deviation; *ESI*, Emergency Severity Index; *km*, kilometers; *IQR*, interquartile range; *mmHg*, millimeters of mercury; *ED*, emergency department; *ACES*, acute care emergency surgery; *MEU*, morphine equivalent unit; *ICU*, intensive care unit; *LOS*, length of stay; *SOFA*, sequential organ failure assessment; *NSAID*; nonsteroidal anti-inflammatory drug.

**Table 2A:** Outcome, defined as pain level at ED departure was greater or equal to 50% of pain level at ED triage.

Categories of Outcome	Result
Pain level at ED departure, mean (SD)	5 (3)
Pain control at departure	
Adequate, N (%)	43 (38)
Inadequate, N (%)	69 (62)
Adequate pain control by admitting medical services (N, % of patient in service) *	
ACES	5 (25)
CS	4 (21)
NS	4 (40)
Soft tissue surgery	6 (38)
Vascular surgery	16 (59)
Other	12 (60)

*ED*, emergency department; *SD*, standard deviation; *N*, number of patients; *ACES*, acute care emergency surgery; *CS*, Cardiac Surgery; *NS*, Neurosurgery.

* Percentage was expressed as number of patients achieving adequate pain control at ED departure as percentage of total patients within one particular service, not the total patient population.

**Table 2B:** Results from multivariable logistic regressions assessing association between clinically-important factors and all patients or only patients who presented with severe triage pain (pain level 8–10). Independent variables were first assessed for association with inadequate pain control at ED departure (Appendix). Independent variables were first assessed for association with inadequate pain control at ED departure (Appendix). Variables with p-value<0.10 were included in the multivariable logistic regression in addition to other clinically significant factors (total MEU, MEU per Kg total body weight, time interval from triage to first administration of narcotics [time to first narcotics]).

Variables	Univariable analysis			Multivariable analysis					
	
	All patients			All patients			Severe pain (8–10)		
		
	OR	95% CI	p-value	OR	95% CI	p-value	OR	95% CI	p-value
ESI	8.29	1.01–68.1	0.049	44	0.00-infinity	0.99	39	0-infinity	0.99
Type of medication	1.39	0.93–2.09	0.10	1.26	0.43–3.70	0.68	1.76	0.4–8.1	0.47
Total MEU	1.03	0.97–1.10	0.34	1.12	0.99–1.26	0.059	1.2	0.99–1.4	0.055
MEU per Kg	0.64	0.27–1.56	0.33	0.22	0.05–0.92	0.037	0.21	0.03–1.2	0.077
Time to first narcotics	1.00	0.99–1.01	0.39	1.00	0.99–1.005	0.55	1.0	0.99–1.004	0.90

*CI*, confidence interval; *OR*, odds ratio; *ESI*, Emergency Severity Index; *MEU*, morphine equivalent unit; *kg*, kilogram.
