# Supplementary material for: Emergency Providers’ Pain Management in Patients Transferred to Intensive Care Unit for Urgent Surgical Interventions
Source: West J Emerg Med. 2018 Aug 8;19(5):877–83. doi: 10.5811/westjem.2018.7.37989 (PMC6123091; doi:10.5811/westjem.2018.7.37989)
Supplement: Supplementary file 1 [file wjem-19-877-s001.docx]

**Appendix.** Results from univariable logistic regressions. Independent variables were first assessed for association with inadequate pain control at ED departure. Variables with p-value ≤0.10 were included in the multivariable logistic regression in addition to other clinically significant factors, that were determined a priori (total MEU, MEU per Kg body weight, Time interval from triage to first administration of narcotics [Tim to first narcotics]).

|  | Unadj. OR | 95% CI | p-value |
| --- | --- | --- | --- |
| Gender | 1.6 | 0.72-3.46 | 0.25 |
| Age | 0.68 | 0.4-1.15 | 0.15 |
| Triage day of week | 0.59 | 0.25-1.41 | 0.23 |
| Triage time of day | 0.69 | 0.32-1.50 | 0.36 |
| Status of teaching hospital | 1.96 | 0.78-4.93 | 0.16 |
| Ground travel distance | 0.85 | 0.60-1.20 | 0.35 |
| ESI | 8.29 | 1.01-68.1 | 0.049 |
| SOFA | 0.97 | 0.51-1.84 | 0.92 |
| Triage SBP | 0.71 | 0.33-1.54 | 0.39 |
| Triage pulse | 1.05 | 0.55-2.02 | 0.88 |
| Triage pain | 0.61 | 0.32-1.19 | 0.15 |
| Need of continuous infusion | 0.73 | 0.32-1.68 | 0.46 |
| ED LOS | 0.98 | 0.87-1.09 | 0.65 |
| Transport type | 0.93 | 0.42-2.08 | 0.87 |
| Admitting services | 0.92 | 0.74-1.13 | 0.42 |
| Number of pain assessment | 0.58 | 0.27-1.25 | 0.16 |
| Type of medication | 1.39 | 0.93-2.09 | 0.10 |
| Total MEU | 1.03 | 0.97-1.10 | 0.34 |
| MEU per Kg | 0.64 | 0.27-1.56 | 0.33 |
| Time to first narcotics | 1.00 | 0.99-1.01 | 0.39 |

*Adj.,* adjusted; *CI*, confidence interval; *ED,* emergency department; *ESI,* Emergency Severity Index; *kg,* kilogram; *LOS*, length of stay; *MEU*, morphine equivalent unit; *OR*, odds ratio; S*BP,* systolic blood pressure; *SOFA,* Sequential Organ Failure Assessment; *Unadj.,* Unadjusted.
